# Sesquiterpene fractions of *Artemisia* plants as potent inhibitors of inducible nitric oxide synthase and cyclooxygenase-2 expression

**DOI:** 10.22038/ijbms.2019.34792.8249

**Published:** 2019-07

**Authors:** Shahrzad Zamani, Seyyed Ahmad Emami, Mehrdad Iranshahi, Shahin Zamani Taghizadeh Rabe, Mahmoud Mahmoudi

**Affiliations:** 1 Immunology Research Center, Bu-Ali Research Institute, School of Medicine, Mashhad University of Medical Science, Mashhad, Iran; 2 School of Clinical Sciences at Monash Health, Faculty of Medicine, Nursing and Health Sciences, Monash University, Clayton, VIC, 3168, Australia; 3 Department of Pharmacognosy, School of Pharmacy; Mashhad University of Medical Science, Mashhad, Iran; 4 Biotechnology Research Center, Mashhad University of Medical Science, Mashhad, Iran

**Keywords:** Artemisia, Asteraceae, Inflammation, Macrophage, Sesquiterpene lactone - fraction

## Abstract

**Objective(s):**

*Artemisia* species are important medicinal plants throughout the world. Some species are traditionally used for their anti-inflammatory effect. The present study was designed to isolate sesquiterpene fractions from several *Artemisia* species and evaluate their anti-inflammatory activities on key mediators and signaling molecules involved in regulation of inflammation.

**Materials and Methods:**

Sesquiterpene fractions were prepared from several *Artemisia* species using the Herz-Högenauer technique. Lipopolysaccharide (LPS)-stimulated J774A.1 macrophages were exposed to isolated fractions. Their possible cytotoxic effect was examined using MTT assay. In addition, nitric oxide (NO) release was measured using Griess method and prostaglandin E2 (PGE2) level was determined by enzyme-linked immunosorbent assay (ELISA). Moreover, protein expression of pro-inflammatory enzymes, inducible nitric oxide synthase (iNOS) and cyclooxygenase-2 (COX-2) were investigated using Western blot analysis.

**Results:**

Nitric oxide level produced by LPS-primed macrophages was significantly decreased with all prepared fractions in a dose-dependent manner. Saturated sesquiterpene lactones-rich species (*Artemisia* *kopetdaghensis,* *Artemisia* *santolina, Artemisia* *sieberi*) showed the highest suppressive activity on NO and PGE2 production via suppression of iNOS and COX-2 expression. Fractions bearing unusual (*Artemisia fragrans* and *Artemisia absinthium*) and unsaturated sesquiterpene lactones (*Artemisia* *ciniformis*) possess less modulatory effect on PGE2 production and COX-2 expression.

**Conclusion:**

It can be concluded that some of the medicinally beneficial effects attributed to *Artemisia* plants may be associated with the inhibition of pro-inflammatory signaling pathways. However, these effects could be dependent on the type of their sesquiterpene content. These findings also introduce new *Artemis* species cultivated in Iran as a useful anti-inflammatory agents.

## Introduction

The genus *Artemisia* belongs the family Asteraceae, which are comprises about 550 species distributed in Europe, Asia, and North America (1). The leaves are very aromatic, alternate, often with long, narrow segments, usually grayish or silvery, hairy. The flower heads are greenish or brownish, without rise. The fruits are seeded, usually flattened or ribbed, without a pappus. The genus in Iran has 34 species which two of them are endemic (2).

Some *Artemisia* species including *Artemisia* *absinthium* L. (Afsantin), *Artemisia* *maritima* L. (Shih), *Artemisia abrotanum* L. (Qaysum Zakar) and *Artemisia dracunculus* L. (Tarkhun) have been used extensively in folk medicine to alleviate several ailments (3). *Artemisia* species exhibit a wide range of leishmanicidal (4), cytotoxic (5-7), anti-microbial (8) and anti-oxidant activities (8). More importantly, the anti-inflammatory effects of *Artemisia* species have been reported (9-11). 

A number of bioactive compounds including acetylenes, coumarins, terpenes, monoterpenes, monoterpene lactones, sesquiterpenes, sesquiterpene lactones, flavonoids, dipeptides, phenolics, coumarin, ethers, esters, esterols and polysaccharides are commonly found in members of the genus (12). In recent years, several sesquiterpenes derived from *Artemisia* including artemisinin and dihydroarteannuin (13), artemisolide and eupatilin (14), scoparone and capillarisin (15), scopoletin (16) have received special attention due to their pharmacological activity on inflammatory processes and other illnesses. 

Nitric oxide (NO) is mainly synthesized by inducible NO synthase (iNOS) which is largely involved in the pathophysiology of many inflammatory diseases (17). Another key enzyme in inflammatory responses is cyclooxygenase-2 (COX-2) which is responsible for prostaglandin E_2_ (PGE_2_) production (18). Several inflammatory stimuli such as bacterial lipopolysachharide (LPS) could activate iNOS and COX-2 expression. Various agents could serve as an important therapeutic target in the treatment of various inflammation-based pathologies (19, 20).

Many medicines commonly used for the treatment of inflammatory diseases could impart various adverse side-effects (21). Numerous researches have focused on herbal cures with lower adverse effects and improved efficacy.

Taking into consideration the above facts, present study aimed to evaluate and compare the anti-inflammatory effects of sesquiterpene fractions isolated from various *Artemisia* species through effects on the production of NO and PGE_2_ as well as on the expression of iNOS and COX-2 by LPS-primed J774A.1 macrophages. To our knowledge, no other study has been carried out to compare the anti-inflammatory effect of *Artemisia* species in regard to their sesquiterpene contents. Aside from this, essentially nothing is known regarding the potential anti-inflammatory property of some *Artemisia* species tested here.

## Materials and Methods

***Chemicals***

Fetal bovine serum (FBS), Dulbecco’s modified Eagle’s medium (DMEM), penicillin and streptomycin were purchased from Gibco Laboratories (Detroit, MI). *Escherichia coli* lipopolysaccharide (LPS, serotype 0111:B4), sodium nitrite, *N*-(1-naphtyl) ethylenediamine, sulfanilamide and 3-(4, 5 dimethylthiazol-2-yl)-2, 5 diphenyl tetrazolium bromide (MTT) were obtained from Sigma (St Louis, MO). ELISA kit for PGE_2_ measurement was bought from Assay Designs (Farmingdale, NY). The protein bands for iNOS and COX-2 were detected with monoclonal antibodies were from Panomics, Inc (Redwood City, CA). Goat anti-rabbit-horseradish peroxide-conjugated antibody was purchased from KOMA Biotech (Seoul, South Korea). ECL-detection reagent was obtained from Amersham (Cardiff, UK).

***Plant materials and isolation of sesquiterpene fractions***

Aerial parts of *Artemisia* species (Table 1) collected from different regions of Khorasan Province, Iran and identified by Dr V Mozaffarian (Research Institute of Forest and Rangelands, Ministry of Jahad Keshavarzi, Iran). Voucher specimens were deposited in the Herbarium of National Botanical Garden of Iran (TARI). The shade dried and powdered plant samples were preserved for further experimentations.

**Table 1:** Tested *Artemisia* species

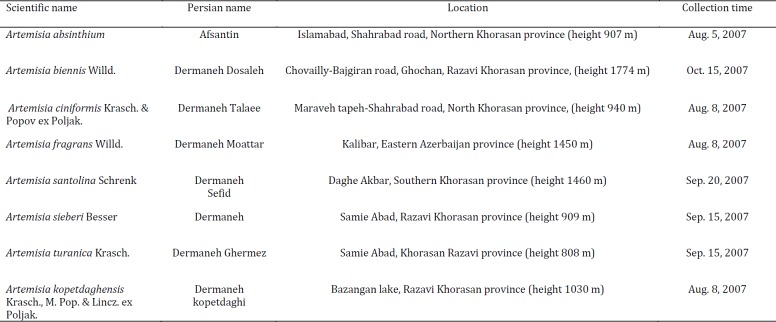

**Table 2:** ^1^H-NMR key data for the sesquiterpene lactone fractions of tested *Artemisia* species

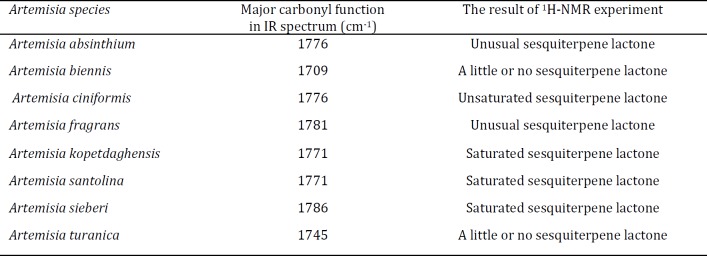

Sesquiterpene fractions were prepared using Herz-Högenauer technique (22). The chlorophyll and common phenolic were removed by lead-(Π)-acetate precipitation and preparing crude sesquiterpene samples for further chromatographic and spectral investigations. Dried and ground plant materials (20 g) were soaked in dichloromethane (DCM; ≈ 100 ml) overnight. The slurry products were then filtered and evaporated *in vacuo*. The gummy residue was dissolved in 96% ethanol (≈ 50 ml) and heated to improve solubility. The aqueous solutions of lead acetate (5%) were included for precipitation of fatty acids, phenolics, and chlorophyll; precipitates were eliminated using filtration through a pad of silica gel (230-400 mesh (Merck, Germany). Lastly, the filtrates were concentrated in a water bath (40-50 °C) until a viscous mass was developed. IR spectra were recorded as KBr disks and in methylene chloride on a Unicam dp 110 spectrometer (Shimidzu Scientific, Japan). ^1^H-NMR (500 MHz) spectra were assessed in CDCl3 by a DRX 500 spectrometer (Bruker, Germany). All fractions were dissolved in dimethyl sulfoxide (DMSO) with the final concentration of less than 0.1% in culture medium.

***Cell culture and treatment***

J774A.1 murine macrophage cell line was obtained from the National Cell Bank of Iran (Tehran, Iran). Cells were cultured in DMEM supplemented with 100 U penicillin/ml and 100 μg streptomycin/ml and 10% heat inactivated FBS at 37 ^°^C in 5% CO_2 _humidified incubator. Cell viability was determined by Trypan blue (0.4% in phosphate-buffered saline [PBS, pH 7.4]) exclusion method. In order to verify their *in vitro *anti-inflammatory effects, J774A.1 cells (1.25 × 10^5^/well) were treated with/without indicated concentrations of fractions (10-100 μg/ml) for 2 hr prior to inflammatory stimulation of LPS (1 μg/ml). The plates were then incubated for additional 24 hr before being used for the cytotoxicity, NO release and PGE_2_ production assays. 

***Determination of cell viability***

Cytotoxicity was examined to verify the possible toxic effect of fractions on macrophages using colorimetric MTT [3-(4,5-dimethylthiazol-2-yl)-2,5-diphenyl tetrazolium bromide] assay (23). Briefly, MTT (5 mg/ml) reagent was added to each well and the plates were then incubated for 3 hr at 37 ^°^C. The formazan crystals formed as a result of MTT reduction in living cells were dissolved in DMSO. The optical density was measured using a microplate reader (Convergent Technologies, Germany) at 545 nm. 

***Measurement of nitric oxide***

Nitrite level, as an indicator of NO synthesis, released into the supernatants of cultured cells was determined using colorimetric Griess assay (24, 25). Briefly, aliquots of cell culture supernatants were incubated with equal volume of Griess reagent, consisting of (1% sulphanilamide in 5% phosphoric acid, 0.1% naphtylenediamine dihydrochloride). The optical density of the mixture was measured after 10 min at room temperature using a microplate reader (Convergent Technologies, Germany) at 545 nm. Nitrite concentrations were calculated by extrapolation from a sodium nitrite standard curve obtained in parallel with sodium nitrite standard solutions.

***Determination of PGE***_2_ ***production***

The amount of PGE_2_ secreted into the culture supernatants was determined using Enzyme-linked immunosorbent assay (ELISA) kit, following manufacturer instructions. Sensitivity of the kit was 16 pg PGE_2_/ml.

***Preparation of whole cellular extract***

Cells were washed twice with cold PBS and lysed in freshly prepared buffer (10 mM HEPES [pH 7.5], 10 mM KCl, 0.1 mM EDTA, 1 mM dithiotheritol (DTT), 0.5% IGEPAL, along with the protease inhibitor cocktail). Cell lysates were then centrifuged at 14,000 g for 3 min at 4^°^ C to obtain the supernatants as total cell extracts. Samples were stored at -70 ^°^C. The protein content in each sample was quantified by use of Bradford assay. 

***Western blot analysis***

Equal amounts of proteins were denaturated in Laemmli sample buffer, separated on 12% SDS-polyacrylamide gel and were then transferred to polyvinylidene difluoride (PVDF) membranes. Non-specific binding sites were blocked with 5% non-fat milk buffer (10 mM Tris-HCl and 100 mM NaCl, pH 7.5) at 4 ^°^C overnight. The blots were incubated sequentially with primary antibodies (anti-mouse iNOS or COX-2 polyclonal antibodies; 1:200 or 1:500 dilution, respectively) overnight at 4 ^°^C. After gentle washing, the blots were incubated with secondary goat anti-rabbit-horseradish peroxide-conjugated antibody (1:10,000 dilution). Immunoreactive protein bands were developed using enhanced chemiluminescence (ECL) detection reagent (Pierce, Rockford, IL). 

***Statistical analysis***

All data were reported as mean±SD of at least three independent experiments. Statistical analysis were conducted using SPSS 11.0 software (Chicago, IL). Significant differences between the groups were determined using one-way analysis of variance (ANOVA). A *P*<0.05 was considered statistically significant.

## Results

***Characterization of isolated sesquiterpene fractions from Artemisia species***

The various IR spectra revealed that all tested samples had notable absorptions between 1730 and 1780 cm^-1^ that point out the existence of carbonyl functional groups. A γ-lactone moiety present as the absorptions > 1760 cm^-1^. Fractions from *A. absinthium*, *A. ciniformis*, *A. fragrans*, *A. kopetdaghensis*, *A. santolina*, and *A. sieberi *exhibited significant absorption peaks at 1776, 1776, 1781, 1771, 1771 m and 1786 cm^-1^, respectively. This could be due to the presence of high amount of sesquiterpene lactones in obtained fractions. Maximum absorption peaks of carbonyl groups in *A. biennis *and *A.turanica *samples emerged at 1709 and 1745 cm^-1^ which could represent the low content of sesquiterpene lactones. The ^1^H-NMR spectra confirmed the presence of unsaturated, saturated and unusual sesquiterpene lactones in the fractions (Table 2).

Sesquiterpene fractions did not affect cell viability J774A.1 cells were exposed to different concentrations of fractions in order to exclude the possibility that the observed anti-inflammatory effects were due to their toxicity. None of the fractions at tested concentrations (10-100 μg/ml) significantly altered cell viability. The maximum percentage of growth inhibition of cells following by exposure to the highest tested concentration (100 µg/ml) of fractions of studied *Artemisia* species were *A. absinthium* 92.49±0.86, *A. biennis *92.74±1.51, *A. ciniformis *90.97±1.70, *A. fragrans *93.25±0.56, *A. kopetdaghensis* 90.40±2.60, *A. santolina* 90.00±2.15, *A. sieberi* 91.54±0.56, *A. turanica *90.97±1.70.

***Effect of sesquiterpene fractions on macrophage NO and PGE***_2_ ***production***

**Figure 1:**
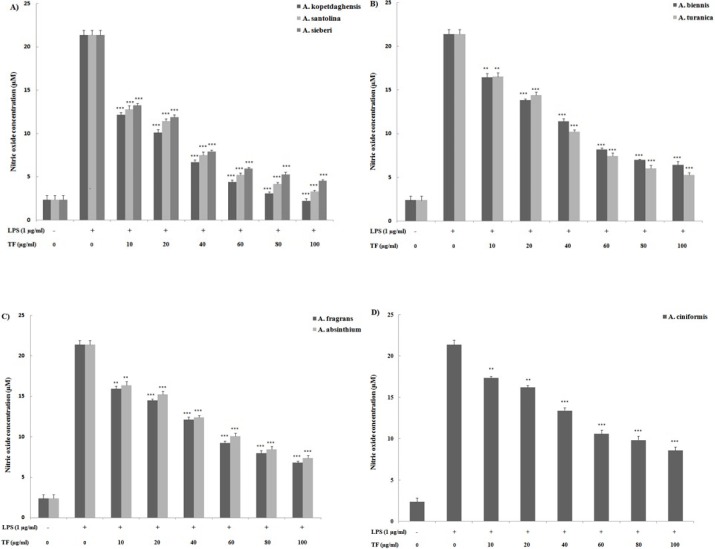
Effect of sesquiterpene lactone fractions of different *Artemisia* species including A) *Artemisia* *kopetdaghensis*, *Artemisia* *santolina*, *Artemisia sieberi*, B) *Artemisia biennis*, *Artemisia* *turanica*, C) *Artemisia* *fragrans*, *Artemisia absinthium*, D) *Artemisia ciniformis* on LPS-stimulated J774A.1 macrophages production of NO. Cells were treated for a total of 24 hr with LPS alone or after an initial 2-hr treatment with the sesquiterpene fractions (10-100 μg/ml). Values shown are means (± SD) of at least three determinations. *P<0.05, **P<0.01 vs LPS alone

To investigate whether sesquiterpene fractions regulate NO production, J774A.1 macrophages were pre-treated with fractions for 2 hr before stimulation with LPS for 24 hr, and nitrite content was measured using Griess reagent assay (Figure 1). Stimulation of J774A.1 macrophages with LPS alone resulted in noticeable upregulation of NO production (21.4±0.5 µM), compared to the unstimulated control (2.4±0.4 µM). However, J774A.1 macrophages pre-treated with sesquiterpene fractions (10-100 μg/ml) displayed a marked dose-dependent reduction in inflammatory LPS-induced NO production. Saturated sesquiterpene lactones-rich species including *A. kopetdaghensis*, *A. santolina*, *A.* *sieberi* most effectively reduced NO release by LPS-activated J774A.1 macrophages.

**Figure 2:**
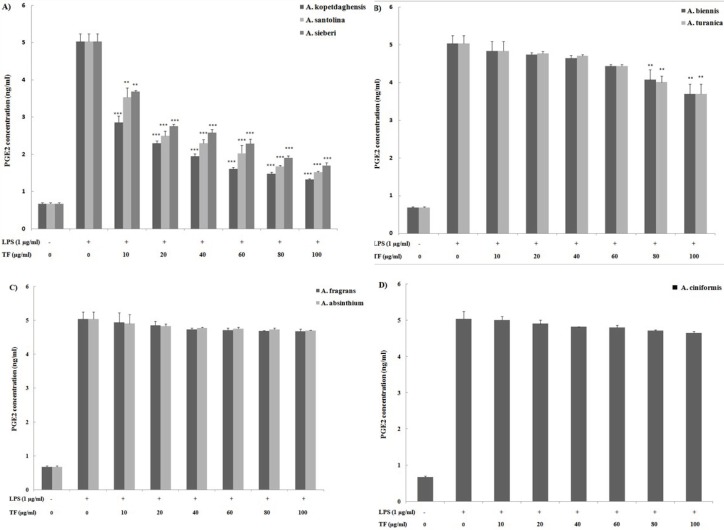
Activitiy of sesquiterpene lactone fractions of various *Artemisia* species including A) *Artemisia* *kopetdaghensis*, *Artemisia* *santolina*, *Artemisia* *sieberi*, B) *Artemisia biennis*, *Artemisia* *turanica*, C) *Artemisia* *fragrans*, *Artemisia* *absinthium*, D) *Artemisia* *ciniformis* on LPS-stimulated J774A.1 macrophages production of PGE2. Cells were treated for a total of 24 hr with LPS alone or after an initial 2-hr treatment with the sesquiterpene fractions (10-100 μg/ml). Values shown are means (± SD) of at least three determinations. *P<0.05, **P<0.01 vs. LPS alone

We next compared the inhibitory effects of fractions on PGE_2_ production as a key mediator of inflammatory responses. The immune response to LPS as an inflammatory stimuli is associated with PGE_2_ production therefore, the effect of sesquiterpene fractions on PGE_2_ production was measured in the supernatants of inflammatory LPS-primed J774A.1 macrophages using ELISA (Figure 2). Under unstimulated condition, macrophages release low levels of PGE_2_ (0.7±0.2 ng/ml); however LPS induced a substantive release of PGE_2_ (5.0±0.2 ng/ml). Sesquiterpene fractions containing saturated sesquiterpene lactones (*A. kopetdaghensis*, *A. santolina*, and *A. sieberi*) and fractions containing a little sesquiterpene lactones (*A. biennis* and *A. turanica*) markedly suppressed the LPS-induced PGE_2_ formation. 

***Effect of sesquiterpene fractions on expression of pro-inflammatory iNOS and COX-2***

**Figure 3:**
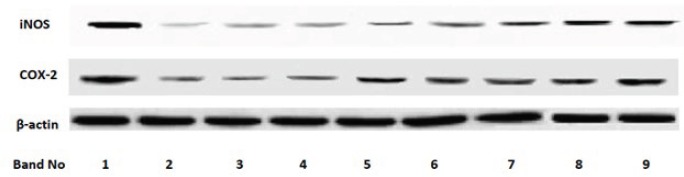
Modulatory effect of sesquiterpene lactone fractions from different *Artemisia* species on LPS-stimulated iNOS and COX-2 protein expression. J774A.1 macrophages were pre-treated (2 hr) with/without 80 μg/ml of fractions and then stimulated for 22 hr with LPS (1 μg/ml). After the end of the 24 hr exposure period, whole cell lysates were prepared and underwent Western blot analysis with antibodies to detect iNOS, and COX-2. Antibody to β-actin was also used to monitor for protein loading. A representative blot is shown as bands 1-9 including: band 1) LPS-stimulated macrophages alone, band 2) *A. *kopetdaghensis, band 3) *Artemisia* *santolina*, band 4) *Artemisia sieberi*, band 5) *Artemisia biennis*, band 6) *Artemisia* *turanica*, band 7) *Artemisia fragrans*, band 8) *Artemisia* *absinthium*, band 9) *Artemisia* *ciniformis*

We further evaluated the effect of sesquiterpene fractions on LPS-induced iNOS and COX-2 expression in J774A.1 cells at the protein level by Western blot analysis (Figure 3). Stimulation with LPS led to increased protein expression (Band 1) in comparison with unstimulated macrophages. However, pre-treatment with tested fractions decreased the levels of iNOS protein expression in LPS-stimulated J774A.1 macrophages (Bands 2-9). Saturated sesquiterpene lactone-rich fractions of *A. kopetdaghensis, A. santolina, *and* A. sieberi *(bands 2-4) exhibited inhibitory activity on the expression of COX-2 protein. This finding revealed that the inhibitory pattern of fractions on iNOS and COX-2 expression overlap with their suppressive effects on LPS-induced NO and PGE_2_ production. 

## Discussion

There is growing attention to natural products derived from medicinal plants. In particular, there is a great interest in their potential use in prevention of inflammatory responses. The use of natural anti-inflammatory products could provide an attractive safe alternative to many common widely-used pharmaceuticals. Along these lines of thought, there are numerous rationale for development of dual iNOS/COX-2 inhibitors as effective anti-inflammatory and analgesic drugs to block the symptoms of inflammation related to nitric oxide and prostaglandin(s) formation due to their increased levels mediate inflammatory responses (26).

Previous phytochemical investigations of *Artemisia* species resulted in the isolation of various classes of chemical compositions (11). Among them, inhibitory effects against inflammatory mediators by several triterpenoids and sesquiterpenes derivatives present in *Artemisia* were widely reported. Although the traditional use of *Artemisia* species as anti-inflammatory agents is supported by scientific reports, to our knowledge, there is little evidence showing specific biological activities of these species. We attempted here to verify the *in vitro* anti-inflammatory potential of sesquiterpene fractions that were isolated from eight *Artemisia* species common to Iran. After preparing the fractions, their anti-inflammatory impact was assessed by measuring any changes in the release of pro-inflammatory mediators, i.e., NO and PGE_2_, produced in response to LPS activation.

The present results demonstrated that each tested sesquiterpene fractions from the isolated *Artemisia* species significantly suppressed LPS-induced NO production in a dose-dependent manner. *A. kopetdaghensis,* *A. santolina*, and* A. sieberi* were revealed to mainly contain saturated sesquiterpene lactones that seemed to be the most potent inhibitors of NO production. Samples from *A. biennis* and *A. turanica *proved to have little or no sesquiterpene lactones in their fractions also dramatically inhibited NO production in the stimulated macrophages. Although *A. absinthium* and *A. fragrans* contained unusual sesquiterpene lactones, they still imparted considerable inhibitory effects as well. Lastly, the sesquiterpene fraction of *A. ciniformis* that mainly contained unsaturated sesquiterpene lactones was also able to inhibit NO production. The Western blot data of iNOS induction were found to be comparable to those from the NO production assays. Taken together, this data suggested to us that the potent anti-inflammatory properties of *A. kopetdaghensis,* *A. santolina*, and* A. sieberi* are mediated in part through the inhibition of the NO synthesis pathway and that this could likely be attributed to the presence of saturated sesquiterpene lactones. However, even though the medium containing fractions was replaced by medium containing LPS, the possibility still also remains that the treatments might have affected subsequent LPS binding itself, i.e., impacting TLR sites on cells.

While the inflammatory mediator prostaglandin E_2_ (PGE_2_) is generated via the cyclooxygenase pathway, it has been reported that NO can directly activate cyclooxygenase pathways and that NO production does not seem regulated by mediators generated by those pathways (17, 18). Assessing the amount of PGE_2_ produced by the treated cells revealed that most of the sesquiterpene fractions failed to inhibit PGE_2_ synthesis or modulate the expression of COX-2 protein. This could be explained, in part, by the fact that changes in PGE_2_ release is most often associated with alterations in COX-2 expression. However, PGE_2_ release was remarkably inhibited by the saturated sesquiterpene lactone-rich fractions of *A. kopetdaghensis*, *A. santolina*, and *A. sieberi* at levels akin to their effects on the production of NO. When both endpoints are examined in tandem, it appeared that the sesquiterpene fractions of *A. kopetdaghensis*, *A. santolina* and *A. sieberi* were apparently the most potent fractions for suppressing NO and PGE_2_ production.

The current findings showed that both iNOS and COX-2 pathways were maximally inhibited by extracts prepared from *A. kopetdaghensis*, *A. santolina* and *A. sieberi*. By comparing these results with those of a previous study (11) in which we reported the anti-inflammatory activity of a sesquiterpene lactone-bearing fraction prepared from *A.* *khorassanica *(SLAK), we surmise that saturated sesquiterpene lactones in various *Artemisia* can impart potent anti-inflammatory effects - in part - through the modulation of both iNOS and COX-2 expression, and that these agents might be classified as dual inhibitors.

## Conclusion

The present study suggested sesquiterpene fractions from *Artemisia* species could be further investigated **to** isolate/identify bioactive compounds that may function as potential anti-inflammatory agents. Studying their modulatory effects in inflammatory animal models would also further validate their potential beneficial properties as natural health products.
